# Supplementary material for: Effective smMIPs-Based Sequencing of Maculopathy-Associated Genes in Stargardt Disease Cases and Allied Maculopathies from the UK
Source: Genes (Basel). 2023 Jan 11;14(1):191. doi: 10.3390/genes14010191 (PMC9859292; doi:10.3390/genes14010191)
Supplement: Supplementary file 1 [file genes-14-00191-s001.zip › genes-2120704-supplementary.pdf]

Supplementary Table S1. (A) Class 3-4-5 variants identified in unsolved probands. **(B)** ABCA4 frequent mild variants identified in unsolved probands. **(C)** Additional sequencing findings in solved probands.

	Patient ID	Gender	Status	Gene	Allele 1		ACMG	Allele 2		ACMG
					cDNA	Protein		cDNA	Protein	
A	4168	M	Unsolved	*CLN3*	c.1141C>T	p.(Arg381Trp)	Pathogenic	+	+	
	4181	F	Unsolved	*RP1L1*	c.3896_3963del	p.(Glu1299Valfs*5)	Likely pathogenic	+	+	
	4756	M	Unsolved	*TIMP3*	c.484G>A	p.(Glu162Lys)	VUS	+	+	
	5152	F	Unsolved	*RP1L1*	c.190C>T	p.(Leu64Phe)	VUS	+	+	
	5202	F	Unsolved	*IFT81*	c.899_900insT	p.(Glu301Argfs*8)	Likely pathogenic	+	+	
	5225	M	Unsolved	*EHMT2*	c.38C>A	p.(Ala13Asp)	VUS	c.38C>A	p.(Ala13Asp)	VUS
B	2272	M	Unsolved	*ABCA4*	c.5603A>T	p.(Asn1868Ile)	MildLP	+	+	
	3528	F	Unsolved	*ABCA4*	c.5603A>T	p.(Asn1868Ile)	MildLP	+	+	
	4181	F	Unsolved	*ABCA4*	c.5603A>T	p.(Asn1868Ile)	MildLP	+	+	
	5859	F	Unsolved	*ABCA4*	c.5882G>A	p.(Gly1961Glu)	Mild	+	+	
C	3670	F	Solved by *BEST1* variants	*ABCA4*	c.5603A>T	p.(Asn1868Ile)	MildLP	+	+	
	5219	M	Solved by *ABCA4* variants	*IMPG1*	c.1157C>A	p.(Ala386Asp)	Likely pathogenic	+	+	
	5258	F	Solved by *CRB1* variants	*RP1*	c.4603C>G	p.(Pro1535Ala)	VUS	+	+	
	5270	M	Solved by *ABCA4* variants	*CACNA1F*	c.40C>A	p.(Pro14Thr)	VUS	+	+	
	5607	F	Solved by *ABCA4* variants	*FBN2*	c.1742A>T	p.(Gln581Leu)	VUS	+	+	
	5608	F	Solved by *ABCA4* variants	*BEST1*	c.1175G>A	p.(Arg392His)	VUS	+	+	
	5857	M	Solved by *ABCA4* variants	*CNGA3*	c.1279C>T	p.(Arg427Cys)	Pathogenic	+	+	
	3656	F	Solved by *ABCA4* variants	*CACNA1F*	c.5585G>A	p.(Arg1862His)	VUS	c.5585G>A	p.(Arg1862His)	VUS
